# Evaluation of Hardfacing Layers Applied by FCAW-S on S355MC Steel and Their Influence on Its Mechanical Properties

**DOI:** 10.3390/ma18153664

**Published:** 2025-08-04

**Authors:** Fineas Morariu, Timotei Morariu, Alexandru Bârsan, Sever-Gabriel Racz, Dan Dobrotă

**Affiliations:** Faculty of Engineering, Victoriei Boulevard, Lucian Blaga University of Sibiu, 550024 Sibiu, Romania; fineas.morariu@ulbsibiu.ro (F.M.); alexandru.barsan@ulbsibiu.ro (A.B.); gabriel.racz@ulbsibiu.ro (S.-G.R.); dan.dobrota@ulbsibiu.ro (D.D.)

**Keywords:** hardfacing, welding, FCAW, mechanical properties, S355MC steel

## Abstract

Enhancing the wear resistance of structural steels used in demanding industrial applications is critical for extending components’ lifespan and ensuring mechanical reliability. In this study, we investigated the influence of flux-cored arc welding (FCAW) hardfacing on the tensile behavior of S355MC steel. Protective Fe-Cr-C alloy layers were deposited in one and two successive passes using automated FCAW, followed by tensile testing of specimens oriented at varying angles relative to the weld bead direction. The methodology integrated 3D scanning and digital image correlation to accurately capture geometric and deformation parameters. The experimental results revealed a consistent reduction in tensile strength and ductility in all the welded configurations compared to the base material. The application of the second weld layer further intensified this effect, while specimen orientation influenced the degree of mechanical degradation. Microstructural analysis confirmed carbide refinement and good adhesion, but also identified welding-induced defects and residual stresses as factors that contributed to performance loss. The findings highlight a clear trade-off between improved surface wear resistance and compromised structural properties, underscoring the importance of process optimization. Strategic selection of welding parameters and bead orientation is essential to balance functional durability with mechanical integrity in industrial applications.

## 1. Introduction

In the context of global industry, the hardness and durability of materials play important roles in improving the performance and efficiency of industrial equipment. Sectors such as aerospace, nuclear, mining, energy, and manufacturing are continuously exploring solutions to extend the lifespan of components subjected to extreme conditions, from high temperatures to heavy mechanical loads. This need goes beyond basic functionality, while also addressing the economic imperative of reducing costs associated with frequent maintenance and replacement of components [1,2].

To improve the wear resistance of materials used across various industrial sectors, advanced welding and cladding methods, such as plasma transferred arc (PTA) welding and flux-cored arc welding (FCAW), are increasingly being adopted. These techniques enhance the mechanical properties of materials, while ensuring excellent bonding of the wear-resistant coatings. Furthermore, advanced processes such as laser cladding and electron beam additive manufacturing (EBAM) enable the optimized customization of technical solutions. Through these technologies, solutions can be precisely tailored to the specific requirements of each sector, thereby maximizing operational efficiency and minimizing material losses due to wear [3,4,5].

The analysis of mechanical properties provides valuable insight into the tensile behavior of clad materials obtained through the welding processes. This analysis reveals challenges directly related to their microscopic structure. Variability in phase distribution and the density of structural defects, such as porosity and inclusions, make predicting the material’s response under stress more difficult. This is a crucial aspect, as performance under both dynamic and static loads determines the long-term reliability of industrial applications [6,7].

Recent research has demonstrated that adding alloying elements to Fe-Cr-C systems significantly alters the microstructure and, consequently, the mechanical properties of the materials. Refinement of carbides and the adjustment of microstructural composition can lead to notable improvements in tensile behavior, providing enhanced strength and durability, which are essential for applications subjected to high mechanical loads [7,8,9,10].

A thorough understanding of the influence of microstructure and alloy composition on tensile behavior is essential. The number of weld layers and their orientation significantly affect the mechanical properties; multiple layers can improve carbide distribution, enhancing the hardness and strength of the material [11]. The thermal process during the welding also plays a crucial role, influencing thermal stability and reducing the risk of residual stresses and distortions that can impair tensile performance. Precise adjustment of welding parameters is vital for maintaining optimal mechanical properties and preventing substrate degradation. These aspects are fundamental for the advancement of hardfacing technologies and their adaptation to the stringent requirements of industrial applications [7,10,12].

In many industrial applications, wear-resistant hardfaced plates are used in the construction of profiled parts manufactured through rolling, which are then integrated into assemblies employed for material transport. The dynamics of material flow and its mass generate specific forces in the direction of movement, affecting these components. Under such conditions, parts fabricated from rolled plate with an additional wear-resistant hardfacing layer are exposed not only to abrasive loads but to tensile stresses as well, while operating the equipment.

Thus, tensile strength becomes a crucial parameter alongside wear resistance. Tensile stresses can significantly influence the structural integrity of components and may lead to premature failures if not properly managed. Considering both types of resistance during the design and manufacturing stages of these plates is essential to ensure their durability and reliability under operational conditions.

While numerous studies have investigated the mechanical performance of hardfaced steel components, particularly those involving Fe-Cr-C systems, a knowledge gap remains concerning the combined effects of weld bead orientation and number of layers on the tensile properties of S355MC steel. Most existing research emphasizes wear resistance or microstructural evolution, but does not address how the mechanical integrity is impacted under multiaxial stress when altering bead direction and layer thickness. This paper presents, for the first time, a comprehensive analysis correlating mechanical degradation with both the orientation of the weld beads (0°, 45°, and 90°) and the number of deposited layers (1 vs. 2), using digital image correlation and 3D scanning to ensure accurate tensile testing. These findings provide new insight into how layer accumulation and orientation influence fracture behavior and process optimization.

## 2. Materials and Methods

To develop effective and durable solutions for such applications, it is essential to carefully select appropriate materials and suitable hardfacing technologies. This selection process plays a critical role in optimizing the combined mechanical and wear-resistant properties of the final product.

The base material used for the deposition of the wear-resistant weld layer is S355MC steel, in accordance with the EN 10149-2/13 standard [13]. It is a hot-rolled steel plate with dimensions of 3000 × 1500 × 4 mm, selected due to its suitable mechanical properties, including a tensile strength of 430–550 MPa and a yield strength of 355–400 MPa. The base material, S355MC steel, has a typical hardness between 140 and 170 HB, which is characteristic for low-alloy structural steels in the hot-rolled condition. The chemical composition of the steel (Table 1) includes elements such as carbon, manganese, silicon, and phosphorus, which are further enhanced with microalloying elements like niobium, titanium, and vanadium. This formulation ensures ideal ductility and strength characteristics, making it essential for efficiently supporting the wear-resistant weld layer.

The flux-cored wire employed in this study has a diameter of 2.4 mm and complies with the EN 14700 T Fe15 classification [14]. Specifically developed for applications that demand high wear resistance, it demonstrates a measured hardness of 62 HRC. The chemical composition analysis, as shown in (Table 2), highlights the presence of carbon and manganese, which provide the necessary hardness to the weld layer. Additionally, elements such as silicon are included to improve the fluidity of the molten metal, while manganese acts as a deoxidizer, strengthening agent, and microstructural stabilizer.

The flux-cored arc welding (FCAW) process was employed for the deposition of protective Fe-Cr-C alloy layers onto the steel substrate, as it is ideal for applying the 2 mm thick coating used in this experiment. The main advantages of this process include high efficiency and the ability to produce dense layers with excellent adhesion to the substrate. The FCAW is characterized by flexibility in controlling the dilution of the base material and allows for precise adjustment of welding parameters to optimize the properties of the deposited layer, such as carbide distribution and size [15].

The FCAW ensures deep penetration and robust adhesion between the deposited layer and the substrate, thereby enhancing the durability of the components. The metal transfer efficiency in the flux-cored arc welding process ranges between 75% and 88% [11]. This high efficiency contributes to achieving deep penetration and strong bonding, which in turn increases the durability of the components. Additionally, the process minimizes porosity and other welding defects, improving the quality and wear resistance of the treated surface. The flexibility in adjusting the welding parameters makes FCAW an adaptable and efficient method, which is widely preferred for enhancing the performance of components operating in demanding environments [11,15].

**Figure 1:**
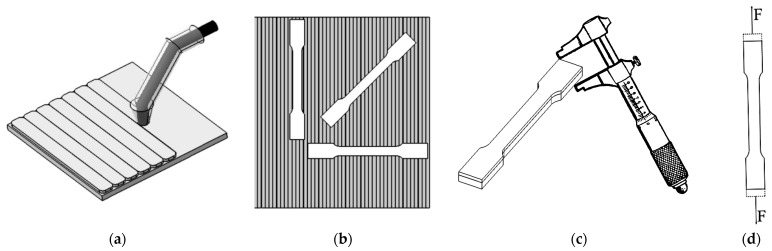
Steps to perform the analysis: (**a**) Specimen welding; (**b**) specimen cutting; (**c**) specimen measurement; (**d**) uniaxial tensile test.

The methodology illustrated in Figure 1 outlines the experimental steps, including the application of the hardfacing layer, either as a single layer or as two successive layers, onto the surface of a 4 mm thick S355MC steel plate serving as the base material. After depositing the wear-resistant layer, the semi-finished product was cut using a waterjet cutting machine, a method chosen for its minimal thermal impact on the material. This technique prevents the generation of excessive heat, thereby avoiding structural alterations or development of residual stresses that could compromise the material’s original properties.

**Figure 2:**
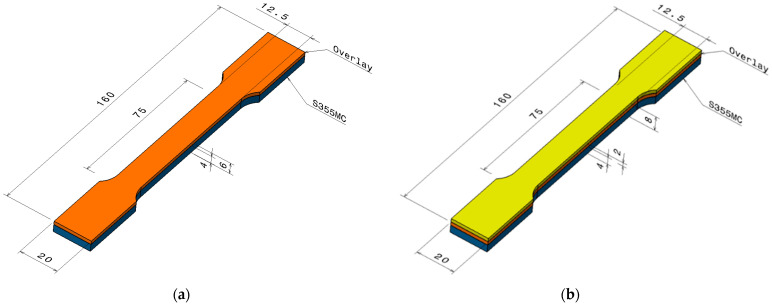
Hardfacing specimen dimensions: (**a**) One layer of weld; (**b**) two layers of weld.

Following the cutting phase (Figure 2), the samples were first subjected to dimensional measurements to ensure accuracy and consistency. Subsequently, a series of tests were conducted to evaluate their mechanical and structural properties, including determination of tensile strength.

**Figure 3:**
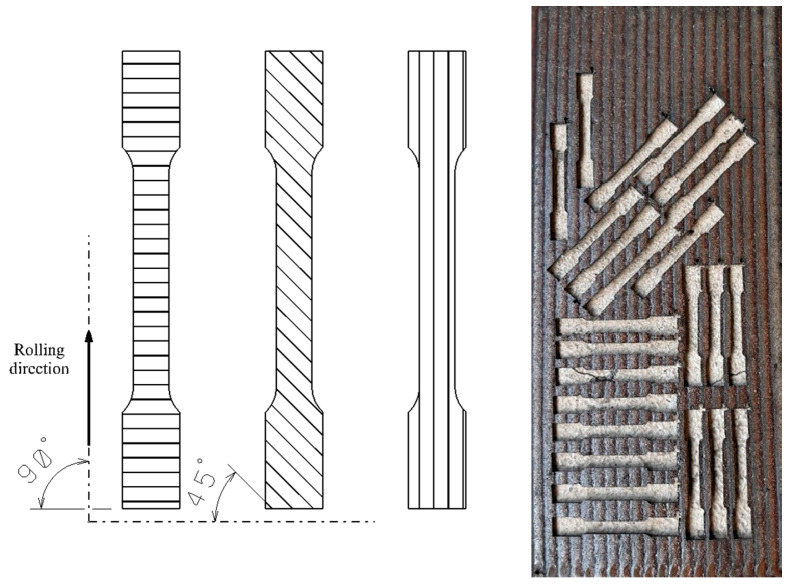
Tensile test specimens oriented according to rolling direction.

In the experiment, the samples were cut based on their orientation relative to the welding direction, as shown in Figure 3. This structured approach was essential for conducting a rigorous comparative analysis of how weld orientation affects the structural and mechanical properties of the material. Samples oriented at 0° were cut so that their length was parallel to the weld bead direction, under the assumption that this alignment could influence the way forces are transmitted and distributed during tensile testing. Those oriented at 45° were extracted at a 45° angle to the weld beads, a configuration chosen to assess the material’s behavior under diagonal stress. Finally, the 90° samples were cut with their length perpendicular to the welding direction, offering insight into the material’s response to transverse forces.

**Figure 4:**
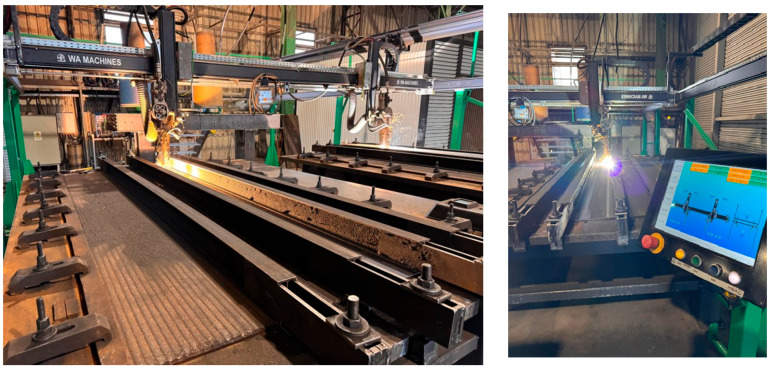
H-Frame welding machine equipped with the D3 Touch control system and oscillating welding heads.

For the application of 2 mm hardfacing layers on the base material, the H-Frame MultiSurfacer machine (Welding Alloys, Cambridge, UK) was used (Figure 4). This equipment, designed for welding process automation, provides efficiency in the application of protective layers and wear-resistant coatings. The automated system combines modular flexibility with advanced technical capabilities, making it suitable for a wide range of industrial applications [16]. The control system enables precise manipulation of welding parameters through a touch-based, intuitive interface, managing multiple modules, including the electrically driven X, Y, and Z axes [17]. It allows the real-time adjustment of welding parameters, offering full control over the process and optimizing weld quality. This level of automation not only improves efficiency but also ensures process repeatability, an essential factor for large-scale industrial production [18,19]. The parameters used for the deposition of weld layers on the base material are detailed in Table 3.

To evaluate the tensile mechanical properties, the tests were conducted using an INSTRON 5587 testing machine (Instron, Norwood, MA, USA), which supports loads of up to 300 kN. This equipment features an advanced data acquisition system that enables the real-time recording of measured values throughout the testing process. All tests were performed in accordance with the ISO 6892-1 standard [20], ensuring that the materials were assessed against relevant industrial performance criteria. The INSTRON 5587 is equipped with a load control system that applied force uniformly and consistently at a testing rate of 15 mm/min. The system was configured to maintain a constant strain rate, thereby minimizing variability and ensuring the reliability and reproducibility of the test results.

**Figure 5:**
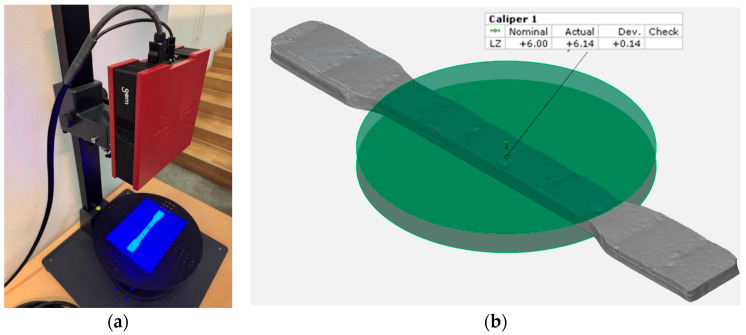
(**a**) 3D Scanning GOM; (**b**) ZEISS INSPECT Optical 3D.

As a part of the experimental procedure involving the testing machine, the average thickness of the samples was determined through the precise three-dimensional measurements. Each specimen was individually scanned using the GOM 3D scanner (ZEISS, Oberkochen, Germany) (Figure 5a), and the resulting data were processed with ZEISS INSPECT Optical 3D software 2023.2.0.1520 (Figure 5b). This approach was crucial for assessing the influence of cross-sectional or thickness variations across the entire calibrated area, where deviations were minimal (0.3 mm). Considering the hypothesis that the cross-section of welded specimens is not perfectly uniform, due to potential cracks or surface irregularities resulting from the welding process, it was decided to introduce the average thickness obtained through 3D scanning as an input parameter in the tensile testing procedure to ensure accurate and representative results.

**Figure 6:**
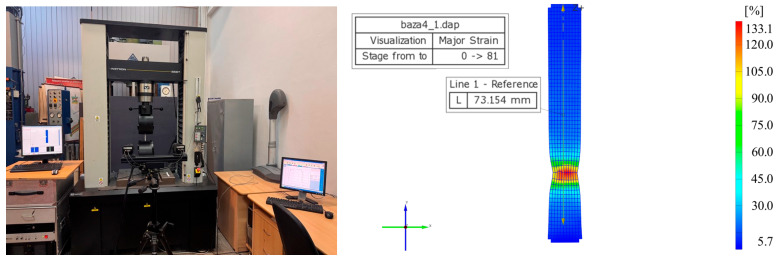
Specific elongation at fracture measured with the GOM-ARAMIS System.

To verify the effect of these variations on the testing results and to ensure the accuracy of the specific elongation at break measurements, the GOM ARAMIS optical deformation measurement system (ZEISS, Oberkochen, Germany) was used (Figure 6). This system enabled detailed capture of deformations within the calibrated area by continuously monitoring two pairs of points distributed along the length of the specimens. Measurements were performed at 0.1 s intervals right before fracture, thereby ensuring precise recording of the material’s behavior under load.

The measurement points were selected to be aligned parallel to the longitudinal axis of the specimens, facilitating coherent and relevant analysis. This rigorous methodology provides a solid foundation for data interpretation and validates the results obtained from the tensile testing machine.

**Figure 7:**
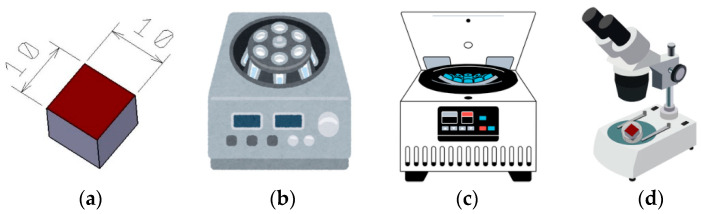
Experimental methodology; (**a**) specimens sectioned to dimensions of 10 × 10 [mm × mm]; (**b**) specimen mounting; (**c**) specimen grinding; (**d**) chemical etching and microscopic analysis.

The microstructural analysis of the deposited weld layers was carried out through the four-stage experimental procedure schematically represented in Figure 7.

The analyzed specimens were extracted from the hardfaced components and sectioned into prisms with a plated surface area of 10 × 10 [mm × mm]. The selected areas for analysis included both the deposited layer and the transition zone toward the base material, to highlight the metallurgical phenomena that occurred during the deposition process. For all metallographic samples, the same extraction approach was applied, with samples taken parallel to the welding direction.

**Figure 8:**
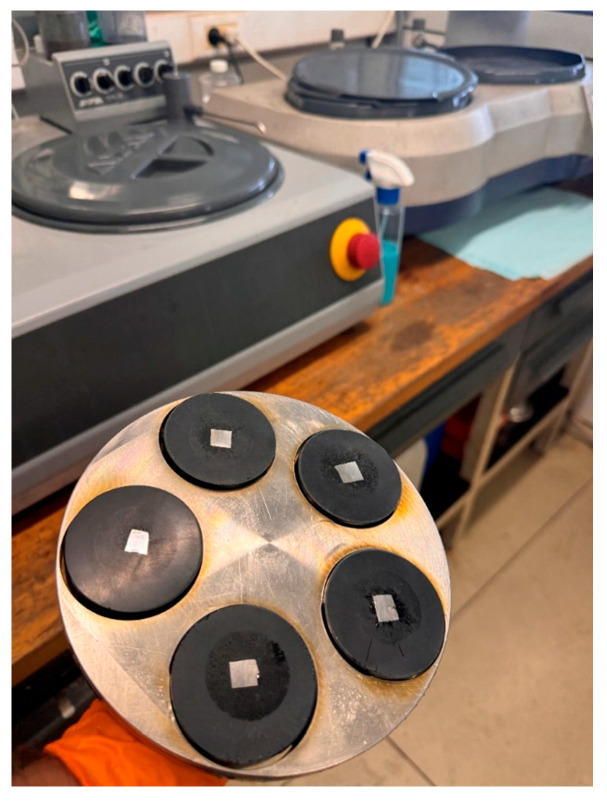
Specimen grinding performed on the ATA Saphir 530 grinding machine.

For the secure fixation and handling during metallographic preparation, the specimens were hot-mounted using the MECAPRESS 3 machine (PRESI, Shanghai, China) (Figure 8).

The parameters used for the hot-mounting process were as follows:

- Temperature: 150 [°C];
- Pressing time: 550 [s];
- Cooling time: 290 [s];
- Applied pressure: 1300 [daN].

The mounting material used was the thermoplastic resin PRESI 00030090 (PRESI, Shanghai, China), which ensured uniform coverage of the specimens and high resistance to elevated temperatures during the subsequent metallographic preparation steps.

To obtain a flat and polished surface, the mounted specimens were subjected to a multi-step grinding and fine polishing process using the Saphir 530 grinding machine (QATM, Mammelzen, Germany). A different abrasive paper was used for each grinding stage. Prior to microscopic examination, the surfaces were chemically treated with Adler’s reagent to reveal the metallographic structures. Using the Leica DM 1750 M metallographic microscope (Leica, Wetzlar, Germany), the microstructure of the samples was examined prior to testing.

## 3. Results

**Figure 9:**
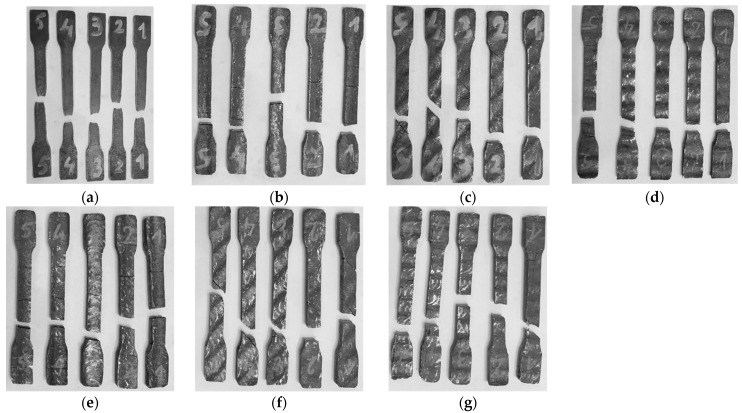
Fractured specimens following uniaxial tensile testing: (**a**) Base material (S355MC); (**b**) hardfaced specimen with one weld layer, oriented at 0°; (**c**) one weld layer, oriented at 45°; (**d**) one weld layer, oriented at 90°; (**e**) two weld layers, oriented at 0°; (**f**) two weld layers, oriented at 45°; (**g**) two weld layers, oriented at 90°.

The experiment involved tensile testing of samples made from S355 steel of a thickness of 4 mm, onto which a 2 mm hardfacing layer was applied by welding, as well as samples made from the same base material with two successively deposited layers. For each type of sample—either with a single weld layer or with two layers—the testing was repeated five times for each orientation strategy (Figure 9). The aim of this research was to determine the impact of the number of weld layers and orientation of the weld beads relative to the rolling direction on the mechanical properties of the material. The testing was carried out in accordance with the ISO 6892-1 standard.

Variations in fracture modes observed among specimens within the same group can be explained by the combined effects of weld bead orientation, the heat-affected zone (HAZ), and microstructural changes caused by the welding process. These factors lead to local differences in mechanical behavior and stress distribution, resulting in different fracture patterns. Additionally, premature fractures near the grips are attributed to internal stress concentrators and microstructural inhomogeneities induced by thermal cycles during welding, which are common in welded specimens and may cause crack initiation outside the expected gauge length.

**Figure 10:**
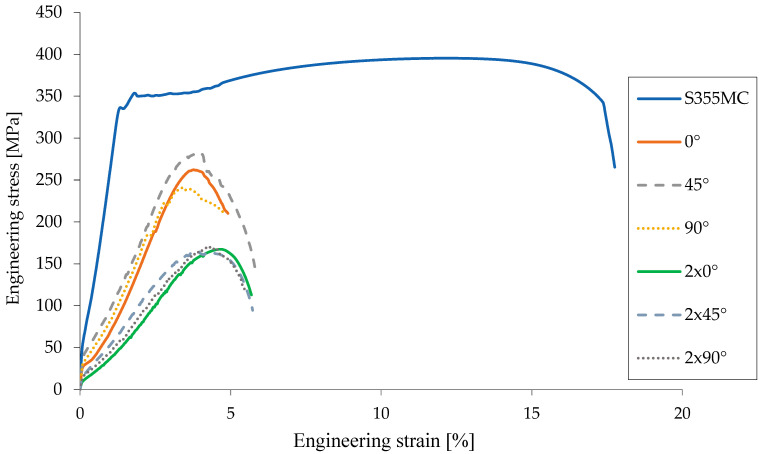
Engineering stress–strain curves for the base material and hardfaced specimens with 1 and 2 layers at 0°, 45°, and 90°.

Figure 10 presents a comparison of the tensile behavior between the S355MC base material and the welded hardfaced specimens oriented at 0°, 45° and 90°, with either one or two weld layers. The figure shows the correlation between the engineering stress (MPa) and engineering strain (%), highlighting variations in mechanical response as a function of the welding configuration and specimen orientation.

**Figure 11:**
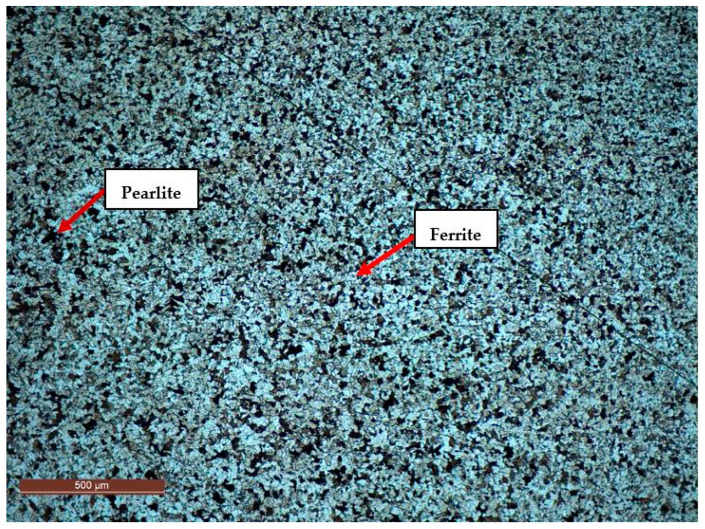
Metallographic analysis of S355 MC material microstructure.

From a metallographic perspective, the analysis of the base material used in this study—S355MC steel (Figure 11)—revealed a microstructure predominantly composed of ferrite and pearlite, a composition typical of low-carbon steels (~0.12% C). This microstructure directly affects the material’s mechanical behavior. Ferrite provides ductility and toughness, enabling good plastic deformability, while the pearlite, though less abundant, enhances yield strength and abrasion resistance through its lamellar structure.

This microstructural characterization is fundamental to understanding the behavior of the deposited layer, as it affects the interaction between the base material and the filler metal within the transition zone. Depending on the thermal cycle applied during the welding and the chemical composition of the filler material, changes, such as grain growth, the formation of intermediate phases, or the diffusion of alloying elements, may occur. All these factors have a direct impact on the adhesion to the substrate and on the mechanical and tribological performance of the deposited layer.

The transition zone between the base material and the deposited layer plays a critical role in determining the mechanical performance and durability of the applied coating. The interface between the two materials is sensitive to thermal and chemical mismatches, which may generate internal stresses that compromise the integrity of the hardfaced layer.

A key factor is the quality of adhesion between the base material and the deposited layer. Insufficient bonding can lead to crack initiation, delamination, or coating detachment, substantially reducing resistance to abrasive wear and corrosion. Therefore, the deposition process must be carefully optimized to prevent such defects and to ensure a stable, long-lasting bond.

**Figure 12:**
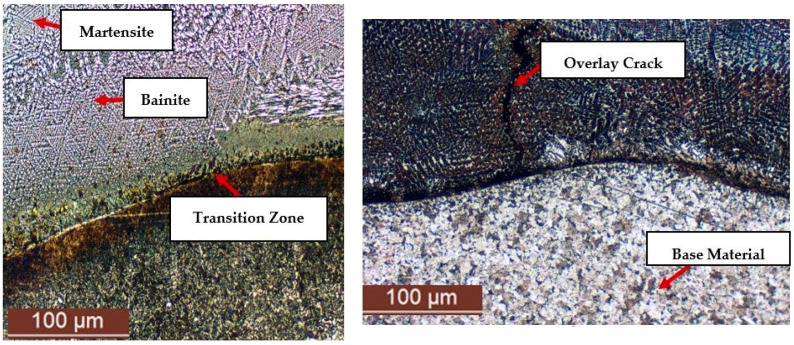
Transition zone showing martensite and bainite morphology for specimens with 1 (**left**) and 2 (**right**) layers. Differences in microstructure are linked to cooling rate and residual stress accumulation, affecting tensile strength and ductility.

The microstructural analysis of the transition zone generally reveals good adhesion between the deposited layer and the base material, with gradual phase transitions driven by chemical diffusion and thermal transfer. A progression was observed from the ferrite–pearlite structure of the base metal to a carbide-rich microstructure in the overlay (Figure 12), which significantly contributes to increased surface hardness.

The diffusion process is intensified by the thermal and chemical gradients generated during deposition, resulting in a well-defined transition zone. The gradual transformation of metallic phases in this region indicates effective fusion between the two materials.

The presence of martensite is associated with rapid cooling, and its grain size is dependent on the cooling rate, directly affecting the hardness and brittleness of the layer. This observation is essential for the optimization of process parameters aimed at achieving an appropriate balance between hardness and toughness.

The examination of the microstructure in the deposited layer, as illustrated in Figure 12, revealed a predominantly martensitic phase in the upper region, along with a significant distribution of chromium carbides. These metallographic features are the result of solidification and phase transformation processes occurring during and after the deposition, directly influenced by the chemical composition of the filler material and the cooling rate of the overlay.

**Figure 13:**
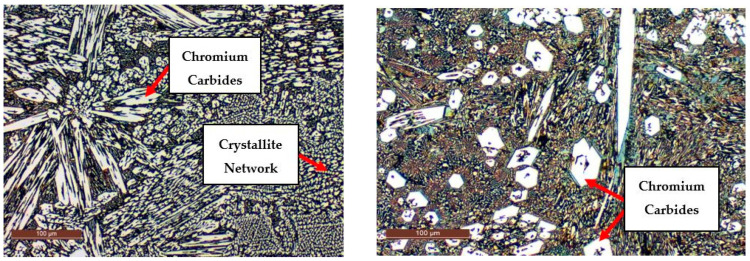
Deposited layer zone showing chromium carbide distribution and porosity (1 layer—**left**). Specimens with two layers exhibit coarser grains and higher porosity, which correlates with the lowest mechanical performance observed in Figure 16 (2 layers—**right**).

The metallographic structure observed in the specimens shown in Figure 13 displays a crystalline network composed of large grains. This grain growth indicates a notable influence of thermal parameters and cooling conditions on the material’s microstructure, which can directly affect its mechanical properties. Additionally, the microstructural analysis revealed the presence of discrete chromium crystallites, suggesting incomplete dissolution of this element into the metallic matrix. This phenomenon is attributed to specific solidification conditions that hindered full chemical homogenization of the material.

As a result, a higher local concentration of chromium was observed in this specimen, which contributed to an increase in hardness. However, the deposited layer exhibited substantial porosity. The presence of pronounced porosity, combined with a non-uniform microstructure, negatively impacted the mechanical strength, reducing the performance of the coating under high-stress conditions.

The uneven distribution of chromium within the metallic structure—found as localized agglomerations of crystallites—indicates incomplete chromium dissolution during solidification. This can lead to significant variations in the microstructure. The presence of such agglomerations may adversely affect mechanical properties, as regions with different Cr concentrations may exhibit variable hardness and inconsistent mechanical behavior. Consequently, the material may contain zones with lower resistance to mechanical loads or wear, thereby compromising the overall performance of the hardfaced layer.

**Figure 14:**
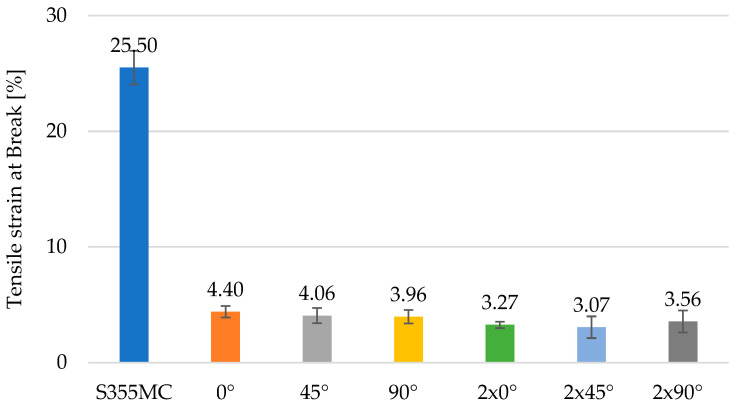
Tensile strain at break for base material and welded specimens.

**Figure 15:**
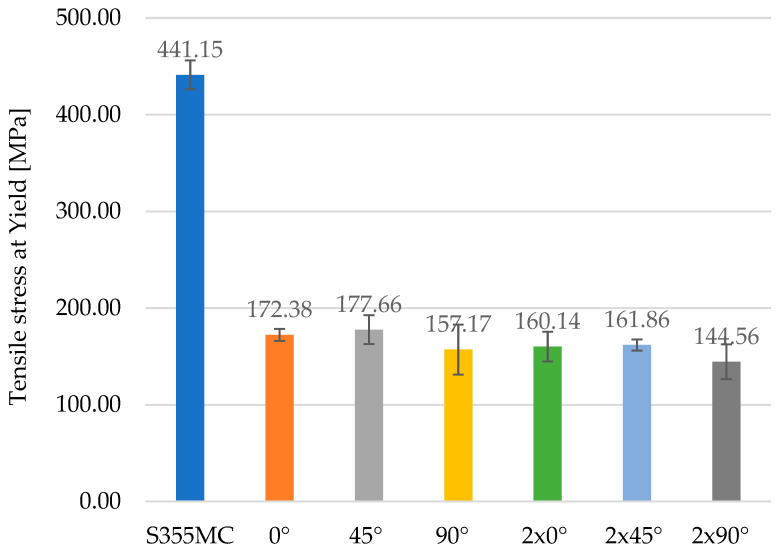
Comparison of tensile yield stress for base material and welded specimens.

**Figure 16:**
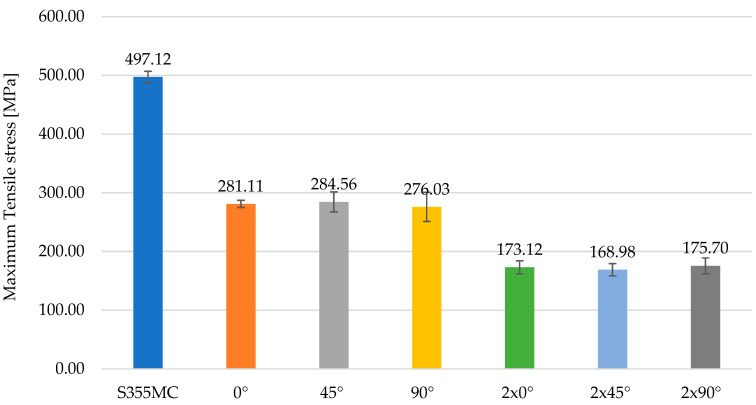
Comparison of maximum tensile stress for base material and welded specimens.

The morphological variability of chromium carbides observed in Figure 12 and Figure 13 directly correlates with the mechanical degradation trends illustrated in Figure 14, Figure 15 and Figure 16. In particular, the denser and coarser carbide network identified in specimens with two weld layers (Figure 13, right) is associated with the lowest tensile strength and ductility values. This microstructural inhomogeneity promotes stress localization, crack initiation, and brittle fracture mechanisms.

Furthermore, the uneven chromium distribution and the presence of undissolved crystallites contribute to local variations in hardness and bonding strength at the interface, explaining the larger standard deviations in specimens tested at 90° orientation. In contrast, more homogeneous carbide morphology in specimens with one weld layer (Figure 13, left) corresponds to a relatively improved mechanical performance. Therefore, the metallographic analysis substantiates the mechanical results by demonstrating the structural effects of layer count and thermal cycling on phase formation, porosity, and carbide dispersion. These factors are critical for interpreting why certain orientations and weld configurations lead to more severe reductions in tensile performance.

## 4. Discussion

The data analysis reveals that the S355MC base material exhibits the highest tensile strength and ductility values, confirming its integrity and robustness in the initial, unaltered state unaffected by the welding processes. In contrast, the specimens welded with a single layer show a reduction in these properties, with relatively minor variations depending on weld orientation, suggesting that the welding process induces degradation of the material’s mechanical performance. Moreover, specimens with two weld layers record the lowest tensile strength and ductility values, indicating a cumulative negative effect of successive weld layer application on the material’s mechanical behavior.

Regarding the specific elongation at break, the S355MC base material demonstrates superior ductility, achieving a significantly higher elongation value of 25.50% compared to the welded specimens, which exhibit considerably reduced values. The samples welded at 0°, 45° and 90° with a single weld layer recorded elongation at break values ranging from 4.06% to 4.40%, while the specimens with two weld layers showed even lower values, varying between 3.07% and 3.56%, as shown in Figure 14. These results indicate that the welding process negatively affects the material’s ability to plastically deform under tensile stress, thereby reducing its deformability.

The graphical representation in Figure 15 of the values of tensile stress at yield compares the yield strength of the S355MC base material to that of the welded variants in different configurations. A significant difference is observed between the values recorded for the unaffected material and those obtained for the welded specimens oriented at 0°, 45° and 90°, with either a single weld layer or two layers. The base material exhibits the highest yield strength, reaching 441.15 MPa, while the welded specimens show a considerable reduction in this property.

The samples welded with a single layer recorded yield stress values ranging from 157.17 MPa to 177.66 MPa, whereas those with two layers exhibited values between 144.56 MPa and 161.86 MPa. These results suggest that the addition of weld layers significantly compromises the material’s ability to withstand loading without permanent deformation.

Regarding the tensile strength, the diagram in Figure 16 presents significant differences between the S355MC base material and its welded variants. The material in its initial state exhibits a considerably higher tensile strength, reaching a maximum of 497.12 MPa, in contrast with the values recorded for the welded specimens, which show a substantial reduction in this property. The specimens welded with a single layer present tensile strength values ranging from 276.03 MPa to 284.56 MPa, while those with two weld layers indicate an even more pronounced decrease, with values ranging between 168.98 MPa and 175.70 MPa. This difference highlights the negative impact of the welding process on the reduction in the steel’s structural capabilities, demonstrating that the successive application of weld layers further amplifies this degradation.

Error bars representing standard deviation were included to assess the data reliability across five repeated tests. The relatively narrow error ranges in most configurations confirm the consistency of the measurement process and validate the observed trends in tensile performance.

The addition of standard deviation bars (±1 SD) in Figure 14, Figure 15 and Figure 16 offers insight into the consistency of test results. For most specimens, variations remained within acceptable ranges (<5% of mean values), confirming the reproducibility of the testing methodology. In configurations involving two weld layers and 90° orientation, greater deviation was observed, attributed to microstructural inhomogeneities and residual stresses induced by heat accumulation during successive welding passes.

The dependency of tensile behavior on bead orientation in FCAW hardfacing processes is insufficiently documented in the context of high-strength, low-alloy steels such as S355MC. This study demonstrates that perpendicular orientation (90°) significantly weakens mechanical performance due to stress localization and crack initiation at the bead–substrate interface. While similar phenomena have previously been conceptualized, this is the first quantified, directionally comparative study involving 3D DIC analysis and multilayer deposition, validating the detrimental role of transverse welds under tensile loads.

While the degradation in tensile properties with increased heat input is known, this study provides a precise quantification of this effect in the context of S355MC steel under FCAW. The results suggest that reducing the number of layers or implementing interpass cooling could mitigate structural degradation. Process improvements may include post-weld heat treatments or tailored thermal cycles to balance wear resistance with mechanical integrity.

The directional dependency of mechanical performance is statistically significant, especially between 0°/45° and 90° configurations. The reduced tensile strength and ductility observed in the 90° specimens are primarily caused by stress concentration along the fusion lines perpendicular to the loading axis. This geometry amplifies the role of microvoids and porosity, facilitating crack initiation. Additionally, transverse welds interrupt the grain continuity and promote delamination tendencies under tensile loads. These findings align with the results from the microstructural analysis and the observed porosity and carbide clustering in the deposited layer. Although the general concept of anisotropy in welded structures has been established, the present study provides a novel quantification specific to FCAW overlays on S355MC steel, supported by empirical testing and digital image correlation.

From a metallographic standpoint, the microstructural configuration of the deposited layer significantly affects its mechanical and tribological behavior. The transition zone between the base material and the deposited layer plays a critical role in the overall performance of the coating, influencing adhesion, local hardness, and resistance to mechanical loading. The gradual transition of metallic phases and the formation of chromium carbides indicate good metallurgical bonding between the substrate and the overlay.

Martensitic structures were consistently observed in the upper regions of the deposited layer, resulting from the rapid cooling characteristic of the FCAW process. This phase contributed to localized embrittlement, depending on the distribution of carbides and the presence of porosity.

The metallographic analysis confirmed that the microstructure of the deposited layer is strongly influenced by the welding parameters, as well as the chemical composition of both the base and filler materials. Achieving optimal mechanical and tribological properties in the hardfaced coating depends on a fine, uniform microstructure with evenly distributed carbides and low porosity.

The mechanical degradation from one to two layers is clearly evidenced by the consistent drop in yield strength and tensile strength. The increased number of weld layers leads to greater thermal accumulation, resulting in the following effects:

- Coarser grain structures (observed in metallography);
- Increased residual stress;
- More pronounced porosity and incomplete chromium dissolution;
- Formation of hard and brittle martensitic structures.

These factors collectively impair the ductility and load-bearing capacity. Therefore, while the effect of heat input is recognized in the literature, the present study details its quantitative impact in a multilayer FCAW configuration on a specific structural steel (S355MC), with experimental controls and layered comparison.

Based on this, process recommendations include the use of interpass cooling, optimization of bead overlap, and possible post-weld heat treatment to reduce microstructural damage in multilayer configurations.

## 5. Conclusions

This paper contributes novel insights into the interplay between weld bead orientation and multilayer deposition on the tensile behavior of hardfaced S355MC steel. For the first time, it quantifies directional and structural effects using a combination of DIC, 3D scanning, and repeat testing. The results demonstrate a clear mechanical penalty associated with transverse bead orientation and successive weld passes, thereby establishing guidelines for process optimization.

The comparative analysis of the graphs regarding the behavior of the S355MC base material and the welded specimens shows a significant decrease in tensile mechanical properties after welding, regardless of the selected configuration. It is evident that the base material, unaffected by any interventions, retains the highest levels of tensile and yield strength as well as superior ductility, illustrating the negative impact of the welding process on the structural integrity of the steel.

Comparing the specimens with a single weld layer to those with two layers, a more pronounced deterioration in mechanical strength is observed in the latter. This phenomenon can be attributed to the accumulation of residual stresses and deeper microstructural alterations due to repeated heat application. By its nature, the welding process induces changes in the metal structure, adversely affecting its initial properties.

Regarding the orientation of the weld beads relative to the rolling direction, the results suggest that specimens oriented at 0° and 45° tend to exhibit a similar mechanical performance, which is slightly superior to that of specimens oriented at 90°. This observation applies to samples with a single weld layer. In the case of samples with two weld layers, the behavior differs, as all such specimens show a performance affected by the thermal effect generated during the second welding pass. This suggests that aligning the welds parallel to the direction of the applied tensile forces may help reduce the negative effects of welding on material strength. In contrast, welding perpendicular to the rolling direction results in the most significant structural alterations and, consequently, the greatest reduction in mechanical properties. This is primarily due to stress concentration at the interface between the weld layers and the base material, which facilitates the crack initiation and propagation under mechanical loading.

It can be concluded that welding and deposition techniques can influence material properties significantly, requiring careful optimization to limit adverse effects on structural integrity. Therefore, the proper selection of welding parameters, including orientation and the number of layers, is essential to balance mechanical performance with the functional requirements of welded components. Future research will focus on implementation of the post-weld heat treatments and alternative filler materials, with the aim of improving ductility and restoring the tensile strength. Further microstructural characterization using SEM and EDS techniques is also planned, to more precisely quantify the role of phase composition and defect distribution in determining mechanical performance.

## Figures and Tables

**Table 1:** Chemical composition of the material S355MC [wt. %].

Mn	C	Ti	Si	S	Al	Nb	P	Fe
0.57	0.06	0.01	0.02	0.01	0.03	0.02	0.01	Balance

**Table 2:** Chemical composition of the overlay 2.4 mm tubular wire [wt. %].

Mn	C	B	Si	Cr	Fe
0.20	5.30	0.30	1.40	29.90	R

**Table 3:** Welding parameters.

Wire Diameter [mm]	Voltage [V]	Current [A]	Wire Speed [m/min]	*Z*-Axis Speed [m/min]
2.4	26	265	2.8	0.2

## Data Availability

The original contributions presented in this study are included in the article. Further inquiries can be directed to the corresponding author.

## Abbreviations

The following abbreviation is used in this manuscript:

FCAW	Flux-cored arc welding.
